# Distribution and genetic variation of hymenolepidid cestodes in murid rodents on the Canary Islands (Spain)

**DOI:** 10.1186/1756-3305-4-185

**Published:** 2011-09-26

**Authors:** Pilar Foronda, Mercedes López-González, Mariano Hernández, Voitto Haukisalmi, Carlos Feliu

**Affiliations:** 1 Institute of Tropical Diseases and Public Health of the Canary Islands, Avda. Astrofísico Fco. Sánchez s/n, 38203 Tenerife, Canary Islands, Spain; 2 Vantaa Research Unit, Finnish Forest Research Institute, PO Box 18, FI-01301 Vantaa, Finland; 3 Laboratory of Parasitology, Faculty of Pharmacy, University of Barcelona, Avda. Diagonal s/n, 08028 Barcelona, Spain

## Abstract

**Background:**

In the Canary Islands there are no previous data about tapeworms (Cestoda) of rodents. In order to identify the hymenolepidid species present in these hosts, a survey of 1,017 murine (349 *Rattus rattus*, 13 *Rattus norvegicus *and 655 *Mus musculus domesticus*) was carried out in the whole Archipelago. Molecular studies based on nuclear *ITS1 *and mitochondrial *COI *loci were performed to confirm the identifications and to analyse the levels of genetic variation and differentiation.

**Results:**

Three species of hymenolepidids were identified: *Hymenolepis diminuta*, *Rodentolepis microstoma *and *Rodentolepis fraterna*. *Hymenolepis diminuta *(in rats) and *R. microstoma *(in mice) showed a widespread distribution in the Archipelago, and *R. fraterna *was the least spread species, appearing only on five of the islands. The hymenolepidids found on Fuerteventura, Lanzarote and La Graciosa were restricted to one area. The *COI *network of *H. diminuta *showed that the haplotypes from Lanzarote and Fuerteventura are the most distant with respect to the other islands, but clearly related among them.

**Conclusions:**

Founder effects and biotic and abiotic factors could have played important role in the presence/absence of the hymenolepidid species in determined locations. The haplotypes from the eastern islands (Fuerteventura and Lanzarote) seem to have shared an ancestral haplotype very distant from the most frequent one that was found in the rest of the islands. Two colonization events or a single event with subsequent isolation and reduced gene flow between western-central and eastern islands, have taken place in the Archipelago. The three tapeworms detected are zoonotic species, and their presence among rodents from this Archipelago suggests a potential health risk to human via environmental contamination in high risk areas. However, the relatively low prevalence of infestations detected and the focal distribution of some of these species on certain islands reduce the general transmission risk to human.

## Background

Cestodes of the family Hymenolepididae (Cyclophyllidea) are ubiquitous and parasites from diverse birds, rodents, insectivores, Chiroptera and some other mammals. According to Czaplinski and Vaucher [1], there are ca. 230 and 620 species of hymenolepidids parasitizing mammals and birds, respectively. Some of the hymenolepidid species of rodents are of health interest, since they are zoonotic and can cause severe diseases in immunosuppressed individuals [2-4].

The Canary Islands are considered a "hot spot" of biodiversity and endemicity of species, including helminths [5]. However, the murid rodents *Rattus rattus *(L., 1758), *Rattus norvegicus *(Berkenhout, 1769) and *Mus musculus domesticus *L., 1758 have been introduced to the Canary Islands.

**Figure 1:**
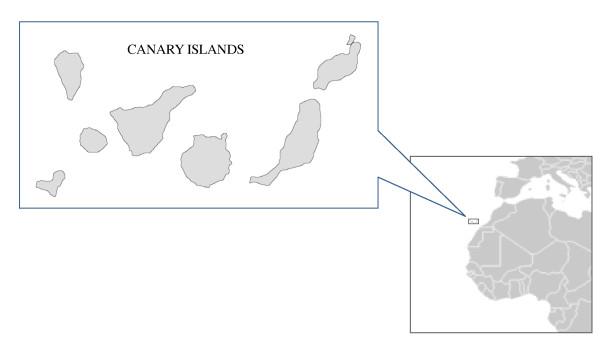
**Location of the Canary Islands**.

The Canarian Archipelago is of volcanic origin, and it is comprised of seven main islands and several islets (Figure 1). The climate varies according to altitude of the islands. Mean temperature and annual precipitation range from about 21°C and 100-300 mm, respectively, in coastal zones, to about 9°C and 500-800 mm, respectively, at higher altitudes. The vegetation is distributed as a function of altitude and orientation. The eastern islands and the lowlands of the higher central and western islands are characterized by dry xerophytic shrub. Temperate forest is located at 300-500 m a.s.l., and the most humid habitat, laurel forest, appears between 550-1300m. A pine forest is the next higher in altitude (1300-2000m) and from here the habitat is constituted by scattered leguminous shrubs.

At present, there is no information about the tapeworms (Cestoda) of rodents on the Canary Islands. The only existing studies on helminths of rodents on these islands concern the nematodes *Angiostrongylus cantonensis *(Chen, 1935) and *Trichuris muris *(Schrank, 1788) [6,7]. In order to identify the hymenolepidid species present in rodents on the Canary Islands, a survey was carried out in the whole Archipelago. The analyses of the distribution of each hymenolepidid species may be used to determine the potential health risks for humans, and to locate the highest risk areas. Furthermore, molecular studies based on nuclear and mitochondrial loci, were performed for hymenolepidid cestodes to confirm the identifications and to analyse the levels of genetic variation and differentiation, given the degree of isolation of the Archipelago with respect to the mainland.

## Methods

The study was carried out on the Canary Islands, located 100 km off the NW coast of Africa, between 13°23´ and 18°8´W and 27°37´and 29°24´N (Figure 1). Since 2007 to 2011, a total of 1,017 murine rodents (349 *R. rattus*, 13 *R. norvegicus *and 655 *M. m. domesticus*) (Table 1) were captured using live-traps on all the islands and on a small islet. The animals were sacrified by cervical dislocation or with CO_2_.

**Table 1:** Prevalences (P) and range of intensities (I) (m = mean intensity) of the hymenolepidid species, *Hymenolepis diminuta, Rodentolepis microstoma *and *Rodentolepis fraterna*, found in rodents from the Canary Islands (Spain)

	*H. diminuta*		*R. microstoma*		*R. fraterna*	
	
	P(%)	I(m)	P(%)	I(m)	P(%)	I(m)
**Tenerife**						
*Rattus *spp. (n = 106)	19.8	1-8 (2.4)	0.9	2	3.7	2-17 (9.5)
*R. r. *(n = 96)	19.8	1-5 (1.9)	1	2	1	17
*R. n. *(n = 10)	20	4-8 (6)	-	-	30	2-15 (7)
*M. m. d.*(n = 111)	-	-	2.7	2-4 (3)	14.4	1-500 (33.2)

**La Palma**						
*Rattus *spp. (n = 18)	33.3	1-7 (2.5)	-	-	5.5	1
*R. r. *(n = 16)	37.5	1-7 (2.5)	-	-	6.2	1
*R. n. *(n = 2)	-	-	-	-	-	-
*M. m. d.*(n = 80)	-	- -	2.5	1-6 (3.5)	3.7	1

**La Gomera**						
*Rattus *spp. (n = 126)	40.5	1-15 (4)	-	-	-	-
*R. r. *(n = 126)	40.5	1-15 (4)	-	-	-	-
*R. n. *(n = 0)	-	-	-	-	-	-
*M. m. d.*(n = 27)	-	-	22.2	1-18 (6.3)	-	-

**El Hierro**						
*Rattus *spp. (n = 53)	18.9	1-20 (3.9)	-	-	1.9	1
*R. r. *(n = 53)	18.9	1-20 (3.9)	-	-	1.9	1
*R. n. *(n = 0)	-	-	-	-	-	-
*M. m. d.*(n = 173)	-	- -	27.7	1-15 (4.7)	-	-

**Gran Canaria**						
*Rattus *spp. (n = 20)	10	1-10 (5.5)	-	-	-	-
*R. r. *(n = 19)	10.5	1-10 (5.5)	-	-	-	-
*R. n. *(n = 1)	-	-	-	-	-	-
*M. m. d.*(n = 41)	2.4	2	-	-	7.3	1-5 (3)

**Lanzarote**						
*Rattus *spp. (n = 20)	5	1	-	-	-	-
*R. r. *(n = 20)	5	1	-	-	-	-
*R. n. *(n = 0)	-	-	-	-	-	-
*M. m. d. *(n = 137)	-	-	5.8	1-32 (14.1)	2.9	1-3 (2)

**Fuerteventura**						
*Rattus *spp. (n = 19)	5.3	15	-	-	-	-
*R. r. *(n = 19)	5.3	15	-	-	-	-
*R. n. *(n = 0)	-	-	-	-	-	-
*M. m. d.*(n = 44)	-	-	2.3	2	-	-

**La Graciosa**						
*M. m. d.*(n = 42)	-	-	7.1	8-21 (14.3)	-	-

**TOTAL**						
*Rattus *spp. (n = 362)	25.4	1-20 (3.4)	0.3	2	1.7	2-17 (6.7)
*R. r. *(n*=*349)	25.8	1-20 (3.4)	0.3	2	0.9	1-17 (6.3)
*R. n. *(n = 13)	0.1	2	-	-	23.1	2-15 (7)
*M. m. d.*(n = 655)	15.4	4-8 (6)	10.8	1-32 (6)	4	1-500 (21.7)

R. r.= Rattus rattus, R. n.= Rattus norvegicus, M. m. d. = Mus musculus domesticus.

The obtained cestode specimens were preserved in 70% ethanol for morphological study and in 100% ethanol or frozen at -80°C for DNA extraction. Cestodes were stained in ferro-acetic carmine, mounted in Canada balsam and studied morphologically and morphometrically with the use of a light microscope. Morphological identification of hymenolepidid cestodes was based on Czaplinski and Vaucher [1]. *Rodentolepis straminea *(Goeze, 1882) from *Apodemus sylvaticus *(L., 1758) from the Pyrenees (France) was also analyzed. Statistical χ^2 ^test with one degree of freedom was used to determine differences in the prevalence of the hymenolepidid species between islands.

### Molecular analyses

Total genomic DNA was extracted for hymenolepidid cestodes using the Fast DNA (BIO 101 Systems) kit, following the manufacturer's instructions and the obtained DNA stored at 4ºC. The nuclear internal transcribed spacer 1 (*ITS1*) region was amplified with the primers F3 5'GCGGAAGGATCATTACACGTTC 3' and R3 5' GCTCGACTCTTCATCGATCCACG 3', previously designed by Macnish *et al. *[8]. PCR amplifications were performed in a total volume of 50 μl, containing 1X buffer (Bioline, London), 0.2 mM of each dNTP, 1 μM of each primer, 1U of Taq DNA polymerase (Bioline, London), 1.5 mM MgCl_2_, and 20 ng of total genomic DNA. PCR conditions were as follow: 2 min at 94ºC followed by 35 cycles of denaturation at 94ºC for 20 sec, annealing at 56ºC for 20 sec, and extension at 72ºC for 30 sec, with a final extra extension step at 72ºC for 5 min.

The mitochondrial cytochrome oxidase 1(*COI*) gene was amplified in two overlapping fragments with primers FCOI 5' TTGAATTTGCCACGTTTGAATGC 3' and RCOI 5' GAACCTAACGACATAACATAATGA 3' [9] and HyCF 5' TATGTTAGACTGAGTGTTTTCA 3' and HyCR 5' TAATACATAAACCTCGGGATG 3', designed by us for this study based on the consensus sequence between different species. For these regions the amplification conditions were: 94ºC for 2 min and 35 cycles of 94ºC for 20 sec, 52ºC for 20 sec and 72ºC for 30 sec, and 72ºC for 5 min as the final extension.

The amplifications were carried out in a Labnet thermocycler (Labnet International, Inc). Amplification products were analyzed on 1.7% agarose gel and visualized by ethidium bromide staining. PCR products were purified using UltraClean PCR Clean-up kit (MO BIO, Carlsbad, CA). Purified PCR products were sequenced at Macrogen Inc. (Korea) and the Genomic Service of the University of La Laguna.

To elucidate any similarities in sequences with those previously published in GenBank, a BLAST search was carried out. Supplemental sequences of hymenolepidid cestodes were obtained from GenBank and added to the alignments. New and previously published sequences were aligned with the multiple alignment program ClustalW as implemented in Mega 4.0 [10] and indels were corrected manually in the *ITS1 *fragment to minimize alignment gaps. Positions corresponding to regions of uncertain alignment were always excluded from the analysis.

Variable positions of the alignment for the *COI *of *H. diminuta *were used to construct a network with the Network 4.6 program [11] using the median joining distance. Because it was not possible to get the same length for all the sequences of *H. diminuta*, the alignment was divided into three parts (344 bp, 403 bp and 389 bp) and three networks were inferred.

Animal trapping and use was approved by the Governmental competent entity "Excmos. Cabildos Insulares" of all the islands.

## Results

Three species of hymenolepidid cestodes were identified, i.e. *Hymenolepis diminuta *(Rudolphi, 1819), *Rodentolepis microstoma *(Dujardin, 1845) Spasskii, 1954 and *Rodentolepis fraterna *(Stiles, 1906), appearing in 9%, 7% and 3.1%, respectively, of all the studied rodents. All three species were found both in rats and mice (Table 1).

### Spatial distribution

*Hymenolepis diminuta *and *R. microstoma *showed a widespread distribution in the Archipelago. *Hymenolepis diminuta *was found on all the seven islands and had a high prevalence in rats, appearing in a quarter of them (Table 1). The rats from La Gomera and La Palma showed the highest prevalences (Table 1) and statistically significant differences compared to the other islands. Moreover, the prevalence on La Gomera was higher than on El Hierro (χ^2 ^= 5.12; *P *< 0.05), Tenerife (χ^2 ^= 4.26; *P *< 0.05), Gran Canaria (χ^2 ^= 5.59; *P *< 0.05), Lanzarote (χ^2 ^= 6.10; *P *< 0.05), and Fuerteventura (χ^2 ^= 5.72; *P *< 0.05). In the case of La Palma statistical differences in the prevalences were found with Lanzarote (χ^2 ^= 4.13, *P *< 0.05) and Fuerteventura (χ^2 ^= 3.84; *P *< 0.05).

*Rodentolepis microstoma *was detected on all the islands, except Gran Canaria. The highest prevalences for this species were detected in mice from El Hierro and La Gomera (Table 1) appearing with percentages significantly higher than in the other islands. Mice from El Hierro were more frequently parasitized than mice from La Palma (χ^2 ^= 17.64; *P *< 0.001), Tenerife (χ^2 ^= 23.62; *P *< 0.001), Lanzarote (χ^2 ^= 20.30; *P *< 0.001), La Graciosa (χ^2 ^= 6.05; *P *< 0.05) and Fuerteventura (χ^2 ^= 10.08; *P *< 0.005). Also the prevalences detected on La Gomera were higher than those on La Palma (χ^2 ^= 10.51; *P *< 0.005), Tenerife (χ^2 ^= 12.69; *P *< 0.001), Lanzarote (χ^2 ^= 7.11; *P *< 0.01), and Fuerteventura (χ^2 ^= 6.75; *P *< 0.01).

Finally, *R. fraterna *was the rarest and least spread species, appearing only on five of the islands and with prevalences lower than 10% except for mice on Tenerife (Table 1) where these hosts were more parasitized by *R. fraterna *than mice from La Palma (χ^2 ^= 5.32; *P *< 0.05) and Lanzarote (χ^2 ^= 10.04; *P *< 0.005).

**Figure 2:**
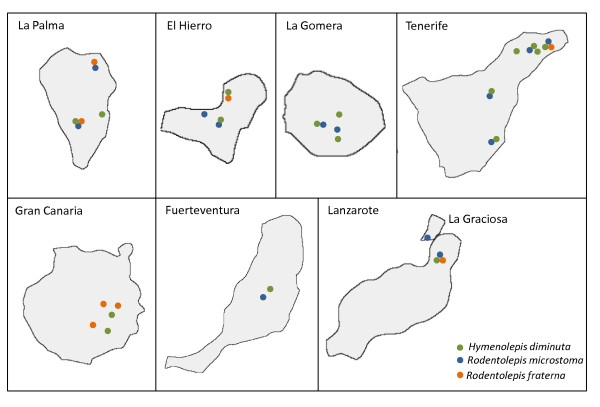
**Distribution of the three hymenolepidid species found in the Canary Islands**.

In some cases, the spatial distribution of the species within islands was not uniform. On Tenerife, *R. microstoma *was detected only in the northeast of the island. In the case of El Hierro, *R. fraterna *was focused to one location called Guinea. The hymenolepidids found on Fuerteventura were located in the central part of the island, while on Lanzarote they were found only in the north. Finally, the only hymenolepidid species detected on La Graciosa islet, *R. microstoma*, was found only in a single peridomestic area (Figure 2).

### Molecular analyses

For the *ITS1 *sequences the fragments analyzed varied in size within species being necessary the inclusion of gaps in order to align the sequences. The longest were 704, 591 and 573 bp for *H. diminuta, R. microstoma *and *R. fraterna*, respectively. In the case of *COI *gene, no indels were observed, within or among the different species to align the sequences, and although partitioned in three fragments, a segment of 1136 bp could be analyzed. All the sequences obtained have been deposited in the GenBank with accession numbers JN258038-JN258041 for the *ITS1*, and JN258042- JN258053 for the *COI *gene.

#### Hymenolepis diminuta

**Figure 3:**
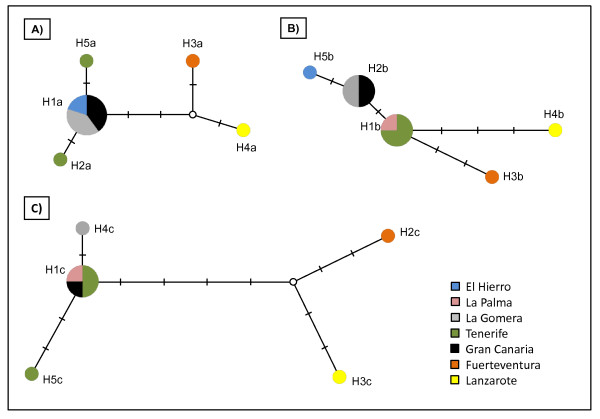
**Median-joining network of the *COI *haplotypes found for the three fragments analyzed for *Hymenolepis diminuta *from Canary Islands**. Circle sizes are proportional to haplotype frequencies and each island is symbolized by a different colour. The small white dots denote unsampled haplotypes and the distances are proportional to the number of mutational changes.

The alignment of 704 bp, for the *ITS1 *sequences of *H. diminuta *from all the seven islands together with a sequence from the GenBank [AF461125] showed only two variable positions. In this alignment two microsatellites were observed, *(TGT)n *and *(GA)n*. For *COI*, six, seven and 11 variable positions out of 344, 403 and 389 bp, respectively were found for the first, second and third fragment, defining five different haplotypes for each region (Table 2). The three networks constructed showed the haplotypes from Lanzarote and Fuerteventura as the most distant with respect to the other islands, but clearly related among them (Figure 3). The fragments 1 and 3 indicated that Lanzarote and Fuerteventura haplotypes seem to have shared an ancestral haplotype turn very distant from the most frequent one that was found in the rest of the islands. Three, three and six mutations at first, second and third fragments, respectively, separated the eastern islands from the central/western islands (Table 2, Figure 3).

**Table 2:** Haplotypes found for each fragment of *COI *gene analyzed for *Hymenolepis diminuta *in Canary Islands.

Haplotypes	Variable positions											Islands						
		1	1	1	2	2												
**Fragment 1 ** (344 bp)	7	0	1	2	8	9						**H**	**P**	**G**	**T**	**C**	**F**	**L**
	6	3	8	4	6	2												
								
H1a	C	A	C	G	A	T						1		2		2		
H2a	T	.	.	.	.	.									2			
H3a	.	.	.	A	G	C											1	
H4a	.	.	G	A	G	.												1
H5a	.	G	.	.	.	.									1			

	4	4	4	5	6	6	7											
**Fragment 2 ** (403 bp)	2	8	8	5	9	9	3					**H**	**P**	**G**	**T**	**C**	**F**	**L**
	7	4	7	0	1	7	6											
								
H1b	A	T	T	C	A	A	T						1		3			
H2b	.	C	.	.	.	.	.							2		2		
H3b	.	.	.	.	G	.	G										1	
H4b	G	.	C	T	.	.	.											1
H5b	.	C	.	.	.	G	.					1						

							1	1	1	1	1							
**Fragment 3 ** (389 bp)	7	7	7	8	8	9	0	0	0	0	0	**H**	**P**	**G**	**T**	**C**	**F**	**L**
	6	7	8	8	9	9	0	2	5	8	8							
	6	8	4	6	2	4	6	0	7	1	7							
								
H1c	C	T	A	G	T	C	C	A	C	T	A		1		2	1		
H2c	.	C	G	T	.	.	T	.	T	.	G						1	
H3c	.	C	G	T	C	.	.	.	T	C	.							1
H4c	T	.	.	.	.	.	.	.	.	.	.			1				
H5c	.	.	.	.	.	T	.	G	.	.	.				1			

H (El Hierro); P (La Palma); G (La Gomera); T (Tenerife); C (Gran Canaria); F (Fuerteventura); L (Lanzarote); bp (base pairs).

#### Rodentolepis microstoma

For the *ITS1*, only sequences for samples from Tenerife, La Gomera and El Hierro islands could be obtained. No variable positions were observed among sequences and the unique haplotype was identical to the sequences from GenBank: AY221159 and AY221166. For the *COI *gene, samples from three islands could be analyzed (Tenerife, La Gomera, and El Hierro). Only one variable position was observed among them, but three positions showed variation with respect to the sequence for this species from USA [12] [GenBank: AB494473].

#### Rodentolepis fraterna

The alignment for the *ITS1 *sequences obtained from samples from Tenerife, La Palma, and El Hierro did not show any variation among them. When these sequences were compared with that deposited in the GenBank [AB494473] only two positions were different. The unique *COI *sequence obtained for one specimen of *R. fraterna *(Tenerife) was identical to a sequence from rats sampled from Egypt [GenBank: GU433103] and differed only in three positions out of 391 bp compared with a sequence obtained from a human sample also from Egypt [GenBank: GU433104].

## Discussion

*Rodentolepis microstoma *and *R. fraterna *have been included within *Rodentolepis *by some authors, but there is still no consensus about their generic position [4,13].

*R. nana *(Siebold, 1852) and *R. fraterna *have sometimes been considered conspecific, but it is still not clear if they are two distinct species, one specialized to man (*R. nana*) and the other (*R. fraterna*) to rodents (see Macnish *et al. *[8]). Moreover, it should be noted that *R. fraterna*, which occurs primarily in *Mus*, has also been found from *Apodemus sylvaticus *based on a molecular study [13]. Macnish *et al. *[8] showed that the representatives of the *nana*/*fraterna *complex from man and rodents are closely related and they form a strongly supported clade with respect to *R. microstoma*. Therefore, taking into account that there is no evidence of a clear genetic or morphological separation between *R. nana *and *R. fraterna*, and the available data in the literature about the life cycle and the health risk are referred to *R. nana*, here we consider them as conspecific.

*Rodentolepis straminea *and *R. microstoma *were earlier considered conspecific [14]. However, Casanova *et al. *[15] suggested independent status for them. The results of Haukisalmi *et al. *[13] fully supported the independent status of *R. straminea *and *R. microstoma *as host-specific parasites of *Apodemus *and *Mus*, respectively, and the present results based on the *ITS1 *sequences also confirm that they are independent species (data not shown) [GenBank: JN258054 for *R. straminea*].

Although *H. diminuta *is more common in rats, in this study one house mouse was parasitized, and the opposite occurred for *R. microstoma*, i.e. although being typical of mice it appeared once in a rat. These exceptional cases have been observed on other islands [16,17].

One of the main results of the present study is the widespread occurrence of hymenolepidid cestodes, particularly *H. diminuta *(in rats) and *H. microstoma *(in mice), in rodents on the Canary Island, despite the varying environmental conditions. Both are also cosmopolitan parasites of their particular hosts, and evidently show a high ability to colonize very different types of environments.

Different hypotheses have been developed to explain the lack of parasite species in introduced hosts, as occurs in the case of *R. fraterna *in La Gomera, Fuerteventura and La Graciosa, and *R. microstoma *in Gran Canaria. Firstly, the founder effect could have played an important role, considering that maybe not all the hymenolepidid species were present in the rodents that invaded the islands. Particularly, the sporadic occurrence of *R. fraterna *may be partly due to its overall rarity, compared with *H. diminuta *and *R. microstoma*, which has increased the probability that the colonizing hosts do not carry this parasite. Secondly, biotic and abiotic factors may be decisive for the establishment of parasite species. These hypotheses have been considered to explain the lack of helminth richness in two other introduced mammal species on the Canary Islands, i.e. the rabbit *Oryctolagus cuniculus *(L., 1758) [18] and the Barbary ground squirrel *Atlantoxerus getulus *(L., 1758) [19].

*Rodentolepis microstoma *was found on Tenerife only in a laurel forest habitat and on El Hierro *R. fraterna *was focused to a single semiarid region. On Fuerteventura, an arid island, *R. microstoma *was found in a farming area only and *H. diminuta *in a place with a very small creek. In the case of Lanzarote, the hymenolepidids were found also only in a farming area, separated from the rest of the island by mountains that could have acted as a geographical barrier. Finally, on La Graciosa although farming, peridomestic and dump areas were sampled, *R. microstoma *was found only close to animal stables.

Complex life cycles requiring multiple host species, is the rule for cestodes. Unique to *R. microstoma *and *R. fraterna *is the capability of reproducing and completing their life cycles without the need of an intermediate host [2]. However, an intermediate host is necessary to complete the life cycle of *H. diminuta*. Several species that act as intermediate host for these three hymenolepidids are present in the Canary Islands, which would facilitate the colonization of these parasites. Appropriate intermediate beetle host species for *R. microstoma *are *Tenebrio molitor *(L., 1758), *Tribolium castaneum *(Herbst, 1797), *Tribolium confusum *(Duval, 1868) (Tenebrionidae) and *Oryzaephilus surinamensis *(L., 1758) (Silvanidae) [20], all being introduced species on the Archipelago [21]. Both *T. castaneum *and *O. surinamensis *are distributed widely and they are considered as invasive species [21].

On the other hand, multiple species have been cited as intermediate hosts of *H. diminuta*, including *T. castaneum *[22], distributed in all of the islands and *T. molitor *[23], present only on La Palma [21]. Burt [24] presented a list of 66 species of intermediate hosts (29 coleopterans, 2 dermapterans, 2 embiopterans, 11 lepidopterans, 9 orthopterans, 11 siphonapterans and 2 diplopods) for *H. diminuta*. This high range could have been one of the main factors in the successful introduction of the parasite to new habitats and regions.

Finally, the most common intermediate hosts capable of transmitting the larval stages of *R. nana *are arthropods, such as the beetle *T. confusum*, which is present on Tenerife, Gran Canaria and Lanzarote, and *T. molitor*, present on La Palma [21,25]. Fleas (Pulicidae), such as *Xenopsylla cheopis *(Rothschild, 1903), which has been introduced to the Canary Islands, is present on El Hierro, Tenerife, Gran Canaria and Fuerteventura (the congeneric species *Xenopsylla guancha *Beaucournu Alcover Launay, 1989, endemic to Lanzarote, may also act as an intermediate host); *Pulex irritans *L., 1758 and *Ctenocephalides *spp., found on Tenerife and Gran Canaria, have also been implicated in the transmission of this parasite [21,25]. The confirmed presence of appropriate intermediate hosts for these hymenolepidid species could explain the establishment of these parasites in the Archipelago.

*Hymenolepis diminuta, R. microstoma *and *R. fraterna*, which are primarily parasites of rodents and secondarily also humans [3,4], have been reported practically throughout the world in places where murid rodents exist. Rodents, particularly rats, are the definitive hosts and natural reservoirs of *H. diminuta *[3], but according to the host-parasite database of the Natural History Museum, London [26], *H. diminuta *has been reported worldwide from ca. 80 species of rodents, and also from insectivores and humans [13]. This suggests that *H. diminuta *includes multiple cryptic species [see 13].

Humans, usually children, can accidentally be infected by ingesting arthropods that are parasitized by larval stages of hymenolepidid cestodes. In developed countries, *H. diminuta *human infection is extremely rare and is limited to rural or degraded areas. Only few hundred human cases of *H. diminuta *have been reported worldwide (see Marangi *et al. *[3] and Tena *et al. *[27]). Evidence of a source of infestation from rats has been found in some of these cases.

*Rodentolepis nana *is the most commonly reported cestode of humans, infecting 175 million people worldwide [28], particularly in the tropics and subtropics [12]. It is more commonly reported as a cause of human infection since its transmission does not require any intermediate host and therefore can be spread directly from person to person or as an autoinfection. It has been shown that infection with *R. nana *can ultimately cause the death of an immunocompromized patient [2].

The presence of the zoonotic species *H. diminuta*, *R. microstoma and R. fraterna *among rodents from the Canary Islands suggests a potential health risk to humans in high risk areas. However, the facts that only *H. diminuta *in *Rattus *spp. shows a relatively high prevalence (Table 1) and that the distribution of some of these species islands is focused in certain islands or habitats, decrease the transmission risk to humans.

### Molecular analyses

The *ITS1 *sequences from *H. diminuta *showed differences in the number of repetitions of both microsatellites. However, considering the high number of copies of the *ITS *in the genome and the fact that all the copies do not have the same repetitions, these differences have not been considered.

The *COI *haplotypes from Lanzarote and Fuerteventrua of *H. diminuta *were the most distant with respect to the other islands, and the possible presence of a common ancestor for both haplotypes, very distant from the most frequent and probably the central haplotype found in the rest of the islands, seem to indicate a separation in time. One or several colonization events could have occurred. If the colonization of the Canary Islands by *H. diminuta *was a unique event, a deep separation of the lineages and a reduced gene flow between the central/western islands with respect to the eastern ones has taken place since the original colonization event. In any case, it would be interesting to confirm if the same distribution pattern is observed in the host species. Therefore, a molecular analysis for the hosts would be highly interesting in order to confirm this hypothesis.

On the other hand, the reduced number of sequences obtained from *R. microstoma *and *R. fraterna*, the low variation and the impossibility to obtain sequences from all the islands, do not allow establishing differences among them. It is relevant the high similarities that the *ITS1 *and *COI *sequences of these two helminths from Canaries show with respect to other parts of the world.

## Conclusions

Three species of hymenolepidids were identified in *Rattus *spp. and *M. m. domesticus *from the Canary Islands, *Hymenolepis diminuta*, *Rodentolepis microstoma *and *Rodentolepis fraterna*. *Hymenolepis diminuta *and *R. microstoma *showed a widespread distribution in the Archipelago, and *R. fraterna *was the least spread species, appearing only on five of the islands. However, the hymenolepidids found on Fuerteventura, Lanzarote and La Graciosa were restricted to a small area. The presence of known intermediate hosts for these cestodes on the islands could have helped in the successful settlement of these parasites.

The fact that haplotypes from Lanzarote and Fuerteventura of *H. diminuta *are genetically the most distant with respect to the other islands, and the possible presence of a common ancestor for both haplotypes, very distant from the most frequent and probably the central haplotype found in the rest of the islands, seem to indicate that two colonization events or a single event with subsequent isolation and reduced gene flow has led to a deep separation. It would be interesting to analyze the hosts in order to confirm the same phenomenon.

## Competing interests

The authors declare that they have no competing interests.

## Authors' contributions

PF, ML and CF performed the field work. CF realized the morphological identification. PF, ML and MH performed the molecular experiments. Discussion of the results was developed by all co-authors. Manuscript was written, read and approved by all the authors.
